# Resting-state high-frequency heart rate variability is related to respiratory frequency in individuals with severe mental illness but not healthy controls

**DOI:** 10.1038/srep37212

**Published:** 2016-11-17

**Authors:** Daniel S. Quintana, Maja Elstad, Tobias Kaufmann, Christine L. Brandt, Beathe Haatveit, Marit Haram, Mari Nerhus, Lars T. Westlye, Ole A. Andreassen

**Affiliations:** 1 NORMENT, KG Jebsen Centre for Psychosis Research, Division of Mental Health and Addiction, University of Oslo, and Oslo University Hospital, Oslo, Norway; 2 Department of Physiology, Institute of Basic Medical Sciences, University of Oslo, Oslo, Norway; 3 Department of Psychology, University of Oslo, Oslo, Norway

## Abstract

Heart rate variability (HRV) has become central to biobehavioral models of self-regulation and interpersonal interaction. While research on healthy populations suggests changes in respiratory frequency do not affect short-term HRV, thus negating the need to include respiratory frequency as a HRV covariate, the nature of the relationship between these two variables in psychiatric illness is poorly understood. Therefore, the aim of this study was to investigate the association between HRV and respiratory frequency in a sample of individuals with severe psychiatric illness (n = 55) and a healthy control comparison group (n = 149). While there was no significant correlation between HF-HRV and respiration in the control group, we observed a significant negative correlation in the psychiatric illness group, with a 94.1% probability that these two relationships are different. Thus, we provide preliminary evidence suggesting that HF-HRV is related to respiratory frequency in severe mental illness, but not in healthy controls, suggesting that HRV research in this population may need to account for respiratory frequency. Future work is required to better understand the complex relationship between respiration and HRV in other clinical samples with psychiatric diseases.

Severe mental illness is associated with a life expectancy almost twenty years shorter than the general population^1^. Increased mortality rates have been largely attributed to cardiovascular disease^2^,^3^,^4^ with sudden cardiac death three times more likely in schizophrenia compared to the general population^5^,^6^. Moreover, rates of sudden cardiac death are even higher in patients using both typical and atypical antipsychotic medications^7^. Cardiorespiratory autonomic dysfunction has been suggested as an underlying mechanism of sudden cardiac death in severe mental illness^8^. The calculation of heart rate variability (HRV), which is the fluctuation of instantaneous heart period over time, provides a non-invasive proximal measure of cardiac vagal modulation^9^,^10^. Originally used as a tool to identify fetal distress^11^, HRV was later adopted to predict the risk of mortality in healthy adults^12^,^13^ and after a coronary heart disease event^14^,^15^,^16^ due to decreased parasympathetic output lowering the threshold for ventricular fibrillation^17^,^18^. HRV has also become central to biobehavioral models of self-regulation and interpersonal interaction as it is a relatively inexpensive means to investigate the relationship between autonomic regulation and behavior^19^,^20^. While early work on HRV primarily used 24-hour Holter monitoring (e.g., refs 21, 22, 23), most recent research within the biobehavioral sciences adopts short-term recordings, typically between 2–5 minutes.

Respiratory parameters (e.g., depth and frequency) are related to heart rate (HR)^24^,^25^,^26^,^27^, which is known as respiratory sinus arrhythmia (RSA). Changes in respiratory patterns can influence both HR and HRV independent of cardiac autonomic activity^24^,^28^. In general, a decrease in respiratory frequency is associated with an increase in the heart period^29^. RSA can be measured by capturing the high frequency (HF) HRV power spectrum that coincides with respiration (typically 0.15 to 0.4 Hz in adults) as the parasympathetic nervous system (PNS) operates using signaling mechanisms that can change HR in phase with respiration^30^. In light of this, we have previously recommended the measurement of spontaneous respiration rate to ensure that vagal modulation does not occur outside the specified HF frequency band^31^. This is especially important to monitor in populations known to have slower (e.g., athletes^32^) or faster (e.g., children and adolescents^33^) respiratory frequencies. Indeed, up to one in five individuals in a sample of healthy participants have been shown to breathe at frequencies slower than 0.15 Hz, which equates to one breath every 6.7 seconds^34^. Including such participants would violate normal cardiorespiratory assumptions if conventional HF-HRV spectral frequency bands are used. As breathing slower than a 0.15 Hz frequency substantially increases the observed power of RSA over that of typical breathing frequencies due to baroreflex recruitment, this confers a sizable impact on spectral HRV measures, highlighting the important link between respiration and HRV calculation.

While it is clear that researchers should monitor spontaneous respiration and control for respiration during tasks and events that can manipulate respiration (for a review, see ref. 31), debate has centered around the need to control for respiration when collecting HRV at rest^35^,^36^. In a series of experiments, Denver and colleagues^35^ demonstrate that respiration is not related to HRV at rest in healthy participants. Relatedly, correcting for respiratory frequency in healthy participants does not appear to provide a better estimate of parasympathetic modulation of heart rate^37^,^38^,^39^. However, this evidence is typically used as justification for not controlling for respiration during resting-state HRV recordings in populations with psychiatric illnesses without a good understanding of cardiorespiratory physiology in these populations. A resting-state period may also, in fact, operate as a modest *stressor* for people with psychiatric illness if they have difficulty sitting still or feel anxious knowing the experimenter is closely monitoring their behavior^40^. Meta-analyses have established an association between poor cardiac autonomic regulation and a range of psychiatric disorders^41^,^42^,^43^,^44^, with most studies not accounting for respiration. Thus, the aim of the present study was to explore the association between respiratory frequency and HRV in a sample of participants with severe psychiatric illness and a non-psychiatric control group.

## Method

The current study was undertaken and reported in accordance with the Guidelines for Reporting on Articles on Psychiatry and Heart rate variability (GRAPH)^40^, which provides a standardized set of criteria for reporting studies examining HRV in psychiatric populations (Supplementary Table S1). The present study is a part of the Thematically Organised Psychosis (TOP) study. Experimental protocols were approved by the Norwegian South-East Regional Committee for Medical and Health Research Ethics (REK sør-øst). Methods were carried out in accordance with the approved guidelines. Written informed consent was obtained from all participants after they received information regarding the study.

### Participants

Interbeat interval and respiration data were analysed from 55 patients with severe mental illness and 149 control participants without mental illness that were at least 18 years old with typical respiratory peak frequencies (i.e., between 0.15 Hz and 0.4 Hz). Data on HRV measures from this sample have been previously reported^45^ (this study reports a larger sample size as respiratory data was not collected from all participants). All included participants were without cardiovascular or metabolic diseases (confirmed by study clinicians). Patients were recruited from psychiatric units at four major hospitals in the Oslo region (*n* = 55) and fulfilled DSM-IV diagnosis of a schizophrenia spectrum disorder (*n* = 34; 72.7% males; schizophrenia *n* = 17; schizoaffective *n* = 4; schizophreniform disorder *n* = 1; other psychosis = 12) or a bipolar disorder spectrum disorder (*n* = 21; 39.1% males; bipolar disorder I *n* = 13; bipolar disorder II *n* = 7; or bipolar disorder NOS *n* = 1). All patients completed The Structured Clinical Interview for DSM-IV Axis I Disorders^46^ and the Positive and Negative Syndrome Scale (PANSS), which indexes the severity of current positive, negative, and general psychopathology symptoms^47^. As we have previously demonstrated that this patient population with schizophrenia spectrum disorders and bipolar spectrum disorders have similar HRV^45^, they were collapsed into one group. Government census records were used to recruit a community representative sample of control participants (*n* = 149; 55.7% males). Clinicians administered the Primary Care Evaluation of Mental Disorders^48^ to confirm that no ongoing psychiatric illnesses were present in control participants. Height and weight were also collected from both participant groups to calculate body mass index (BMI).

### Collection and analysis of physiology data

Five minutes of pulse oximetry data from a functional magnetic resonance imaging (MRI) scanning sequence were used to collect interbeat intervals (IBIs). Data was collected using a photoplethysmograph placed on the right index finger (50 Hz). Participants were supine and instructed to lie as still as possible with their eyes open during the scan, with testing occurring during various times of day. Approximately seven minutes of data were collected in total, however, the first two minutes of data were removed to account for habituation to the imaging procedure. Pulse oximetry data offers an especially accurate approximation of interbeat intervals^49^,^50^. Consistent with recommendations^10^, the raw pulse data was upsampled to 1000 Hz using spline interpolation to refine the pulse peak point to calculate HRV in ARTiiFACT^51^. An algorithm developed by Berntson and colleagues^52^ was used to detect artifacts (e.g., movement). Detected artifacts were visually inspected, with the observer blind to participant group. Approximated IBIs by means of cubic spline interpolation replaced observed artifacts. Absolute high frequency (HF; 0.15–0.4 Hz) power, which represents cardiovagal activity^10^, was computed using the Fast Fourier Transformation (FFT) to assess HRV. The FFT applied a Hanning window of 256-s width with an interpolation rate of 4 Hz (spline interpolation) and an overlap of 50% to the resampled and detrended data (method of least squares). The square root of the mean squared differences of successive heart periods (or root mean square successive differences: RMSSD) was also calculated. RMSSD is a time domain HRV measure that is strongly associated with HF-HRV (*r* = 0.93)^53^, but may be less influenced by respiratory frequency^54^. Finally, absolute HF and RMSSD values were log transformed to better approximate a normal distribution. A chest strain gauge was used to measure respiratory frequency. The Sigview software package (http://sigview.com) was used to compute respiratory frequency. Strain gauge signals were manually checked for artifacts (e.g., signal loss) and peak respiratory frequency within the respiratory band (0.15–0.4 Hz) was computed using the FFT.

### Statistical analysis

All statistical tests were conducted using the R statistical software package^55^. Continuous demographic (e.g., age, BMI) and cardiorespiratory variables were compared with Welch’s *t*-test, and Hedges’ *g* were calculated as a measure of effect size using a custom script (https://github.com/Lakens/perfect-t-test). Gender distributions in the patient and control groups were compared with a chi-squared test. Bayesian Pearson correlation tests were calculated to assess the relationship between respiration and both HRV and HR as they can provide evidence for both the alternative and null hypotheses and produce probabilities that are easily interpretable and less dependent on sample sizes (in comparison to p-values generated from null hypothesis significance tests). The Bayesian Pearson correlation test^56^ assumes a bivariate *t* distribution, which is less sensitive to outliers^57^ than the bivariate normal distribution, which is assumed in frequentist Pearson correlation tests. The probability that these correlations are different was computed by examining the posterior difference in correlations between groups. Frequentist Pearson correlations were also calculated. Finally, differences in respiratory rate and HRV between patients taking antipsychotic medications with a known anticholinergic effect (i.e., olanzapine, quetiapine, and clozapine^58^) were also compared using Welch’s *t*-test. The R analysis script is available at https://osf.io/6wba8/.

## Results

**Figure 1:**
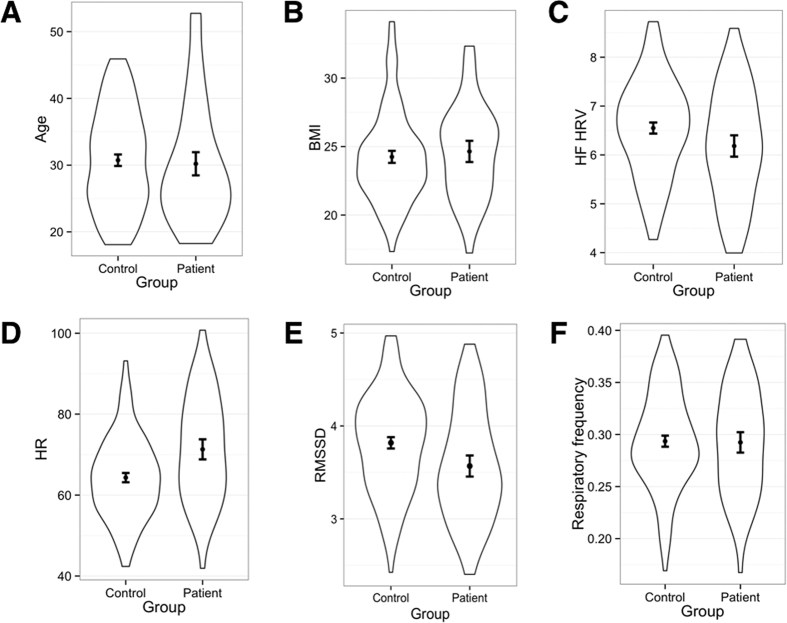
Violin plots with means and 95% confidence intervals for demographic and cardiorespiratory variables. The following variables are shown for the clinical and control groups: age (1A), BMI (1B), HF HRV (1C), HR (1D), RMSSD (1E), and respiratory frequency (1F). Violin plots illustrate the distribution of data by showing the probability density of the data at different values. BMI = Body mass index; HF HRV = Absolute high frequency HRV, log transformed; HR = Heart rate.

Continuous demographics, clinical, and cardiorespiratory variables for each group are shown in Table 1 and Fig. 1. There was no significant difference in age, BMI, and respiratory frequency between groups. A chi-squared test also revealed no difference in the gender distribution between the two groups, χ^2^(1) = 0.02, *p* = 0.87. Average HR (Fig. 1D) was significantly higher in the patient group (Table 1). HRV (Fig. 1C), and RMSSD (Fig. 1E) were significantly reduced in the patient group (Table 1). Continuous demographics, clinical, and cardiorespiratory variables for each broad diagnostic entity (i.e., schizophrenia spectrum disorders vs. bipolar spectrum disorders) are presented in Table 2. There were no significant differences in demographic or cardiorespiratory variables. However, as expected the schizophrenia spectrum disorders group reported significantly greater symptom severity according to the PANSS total score and PANSS subscales (Table 2).

**Figure 2:**
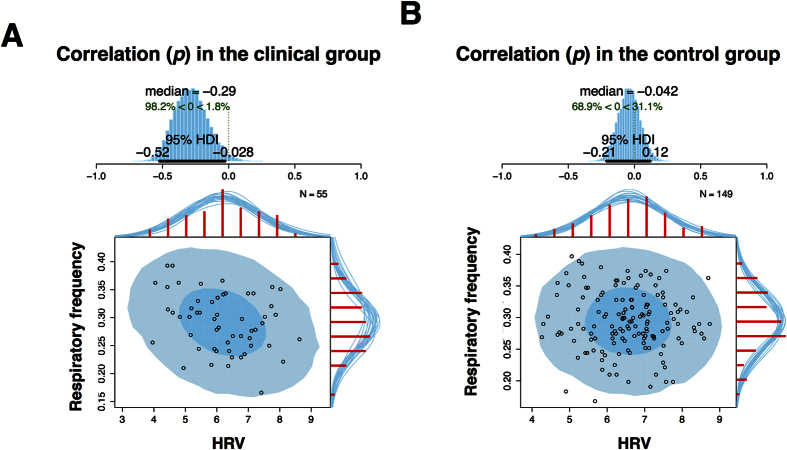
The relationship between respiration and HRV. Plots demonstrate the relationship between respiration and HRV for the clinical (2A) and control group (2B). The blue histograms at the top of each figure show the posterior distribution for the correlation *p* with a 95% highest density interval (HDI). The scatterplots illustrate the relationships between these two variables, with superimposed posterior predictive distributions. The larger light blue ellipse shows the 95% highest density region while with smaller dark blue ellipse shows the 50% highest density region. The red histograms on the top x-axes and right y-axes show the marginal distributions of the data drawn from the posterior. HDI = Highest density interval.

The Bayesian Pearson correlation test revealed an estimated correlation (*p*) of −0.29 between HF-HRV and respiration in the patient group [95% CI (−0.53, −0.03), n = 55; Fig. 2A], suggesting that the correlation coefficient is less than 0 by a probability of 98.2%. The corresponding correlation (*p*) for the control group was −0.04 [95% CI (−0.21, 0.12), n = 149; Fig. 2B], indicating that the correlation coefficient is less than 0 by a probability of 68.9%. Computing the posterior difference of *p* between these two tests revealed a 94.1% probability that *p* was more negative in the clinical group compared to the control group. The estimated correlation (*p*) between RMSSD and respiration was −0.13 [95% CI (−0.38, 0.15), n = 55], suggesting that the correlation coefficient is more than 0 by a probability of 81.2%. The corresponding correlation (*p*) for the control group was 0.1 [95% CI (−0.07, 0.25), n = 149], indicating that the correlation coefficient is more than 0 by a probability of 87.2%. Computing the posterior difference of *p* between these two tests revealed a 91% probability that *p* was more negative in the clinical group compared to the control group.

The estimated correlation (*p*) between HR and respiration in the patient group was 0.3 [95% CI (0.04, 0.54), n = 55], suggesting that the correlation coefficient is more than 0 by a probability of 98.3%. The corresponding correlation (*p*) for the control group was −0.02 [95% CI (−0.19, 0.14), n = 149], suggesting that the correlation coefficient is less than 0 by a probability of 60.2%. Computing the posterior difference of *p* between these two tests revealed a 96.1% probability that *p* was more positive in the clinical group compared to the control group. Frequentist Pearson correlations provided almost equivalent similar results for all four relationships (Table 3). There was no significant difference in HF-HRV [*t*(39.98) = −0.78, *p* = 0.44; Hedges’ *g* = −0.22], RMSSD [*t*(35.75) = −0.55, *p* = 0.58, Hedges’ *g* = −0.15], or respiratory frequency [*t*(33.56) = −0.9, *p* = 0.38, Hedges’ *g* = −0.25] between individuals in the clinical group taking antipsychotics with anticholinergic properties and those that were unmedicated or taking antipsychotics without appreciable anticholinergic properties.

## Discussion

The present study provides evidence that resting-state HF-HRV is negatively related to respiration frequency in severe psychiatric illness. Consistent with some prior research^35^, there was no significant association between respiration and HF-HRV in a representative healthy control group that was matched for age and BMI. Moreover, a statistical comparison of these two correlations revealed a 94.1% probability that the negative relationship observed in the clinical group is stronger than the corresponding relationship in the healthy control group. In contrast, RMSSD was not significantly associated with respiratory frequency in either group, suggesting it may serve as a more accurate measure of cardiac vagal influence in samples that include both healthy subjects and those who suffer from psychopathology. This is consistent with prior work suggesting that RMSSD may be less susceptible to variations in respiratory frequency^54^. RMSSD calculation methods, which involve the comparison of successive beat-to-beat changes (in contrast to power spectrum analysis), may underlie these observed differences. As vagal influences occur within one second^59^, variations in adjacent RR intervals are thought to exclusively represent exclusive vagal effects^54^ (but see ref. 60). Thus, modest sympathetic contributions to HF-HRV^61^ in the patient group could have contributed to the observed relationship between respiratory frequency and HF-HRV. However, this cannot be examined in the present study as a measure approximating sympathetic nervous system activity (e.g., the pre-ejection period) was not included and sympathetic blockade was not performed. While research has shown that respiratory frequency is not related to HRV during sympathetic blockade^62^,^63^, research has yet to adopt this approach in a population with severe mental illness.

There are four factors that contribute to most of the reductions of HF-HRV^37^; reduced basal firing rate of Nucleus Ambiguus (NA) motor neurons (i.e., IBI interval variation), lower respiratory depth, increased respiratory frequency^64^, and lower central respiratory drive^65^. Under basal conditions in healthy individuals, these four factors converge to explain almost 80% of the variance in HF-HRV^37^. Importantly, NA motor neuron firing provides the strongest contribution, and correcting this for respiratory frequency does not appear to add additional explanatory power^37^,^38^,^39^. While speculative, we suggest that these observed contributions in healthy individuals might dissociate during resting-state in populations that have a higher risk of cardiovascular disease, such as severe mental illness. Moreover, hyperventilation in patient groups (which can occur without changes in breathing frequency^66^) due to state and trait anxiety^67^,^68^ can influence central respiratory drive, which contributes to HF-HRV in addition to PNS outflow. Thus, future research is required to investigate the role of ventilation and respiratory depth in clinical populations to better characterize the link between respiration and HF-HRV.

The present study raises important questions about the degree to which resting HF-HRV reflects cardiac efferent vagal activity in different populations. The results suggest that respiration plays a more prominent role confounding HF-HRV as measure of resting cardiac vagal modulation among those with severe mental illness. Future experimental work using pharmacological autonomic blockade may better characterize this relationship. For instance, propranolol can be used to block sympathetic beta-adrenergic influences on HR (as per prior research^62^) during the manipulation of respiratory rate^69^ to help rule out sympathetic effects. Comparing HF-HRV between a healthy control and severe mental illness group would allow researchers to assess the importance of respiration on vagal modulation. As vagal and sympathetic beta-adrenergic effects are almost the exclusive source of resting-state HR variations, a complementary approach to better understand this observed relationship would be to also compare the resting heart period before and immediately after pharmacological vagal blockade.

A number of potential confounds may contribute to the present results. For instance, anticholinergic medications may have played a role considering their impact on HF-HRV; however, the data suggest that respiratory frequency and HF-HRV are not significantly different in between those taking anticholinergic medications and others. Relatedly, almost one in three individuals with severe mental illness also fulfil the diagnostic criteria for metabolic syndrome (MetS)^70^, which is a collection of symptoms – insulin resistance, increased blood pressure, visceral adiposity, elevated triglyceride levels, and reduced high-density lipoprotein cholesterol levels – that contribute to a greater risk of developing CVD and diabetes^71^. While the exact composition of MetS in the present sample is unknown, it is conceivable that MetS or MetS risk factors may have contributed to the observed results. There are also a number of study limitations worth mentioning. Firstly, additional factors that are known to influence HF-HRV, such as respiratory depth and central respiratory drive, were not collected in the present study. These measures may have been able to account for the observed differences between the clinical and control groups. Relatedly, fitting the stain gauge around the chest may have offered less signal accuracy for participants who tend to breathe diaphragmatically. Second, the study sample size was not large enough to examine the link between HF-HRV and respiration in schizophrenia and bipolar disorder subtypes so it is unclear if a specific clinical population was exerting a disproportionately large influence on the observed results. Regardless, demographic and cardiorespiratory characteristics were comparable between subtypes so any potential effects may have been limited. Third, measures of physical activity were not collected so the role of sedentary behavior (despite comparable BMI) on the observed group differences is not well known. Finally, while pulse oximetry offers an accurate approximation of interbeat intervals, it can difficult to identify cardiac dysrhythmia using this approach compared to ECG examination.

There is no consensus for the best approach to adjust HRV for respiratory frequency. Some alternatives are available to experimentally control for respiration. Firstly, investigators can simply examine their data for relationships between respiration and HRV. Second, a within-subjects regression approach where the HF-HRV is residualized against respiration to partial out its effects can be used^62^. Third, all participants can have their respiration paced to a common cue (e.g., audio tone or visual signal). Fourth, investigators can measure a participant’s spontaneous breathing rate and then use this rate for pacing respiration^72^. Altogether, the present study provides preliminary evidence that researchers may need to account for respiration frequency when calculating HRV in psychiatric samples. Future work is required to characterize the complex relationship between respiration and HRV in non-healthy populations and whether the dissociation of systems underpinning autonomic cardiac control contributes to increased CVD risk in these groups.

## Additional Information

**How to cite this article**: Quintana, D. S. *et al.* Resting-state high-frequency heart rate variability is related to respiratory frequency in individuals with severe mental illness but not healthy controls. *Sci. Rep.* **6**, 37212; doi: 10.1038/srep37212 (2016).

**Publisher’s note**: Springer Nature remains neutral with regard to jurisdictional claims in published maps and institutional affiliations.

## Supplementary Material

Supplementary Information

## Figures and Tables

**Table 1:** Demographic, clinical, and cardiorespiratory variables.

Group	HC (n = 149)	PSD (n = 55)	*t* (df)	*p*	Hedges’ *g*	95% CI
Age	30.74 (7.52)	30.2 (9.08)	−0.39 (82.81)	0.76	−0.07	−0.37; 0.25
BMI^a^	24.25 (3.32)	24.64 (3.39)	0.63 (65.26)	0.53	0.12	−0.25; 0.48
HF-HRV	6.65 (0.99)	6.18 (1.14)	−2.11 (85.74)	0.04	−0.35	−0.64; −0.02
RMSSD	3.82 (0.53)	3.57 (0.59)	−2.74 (87.89)	0.01	−0.45	−0.74; −0.12
Heart rate (bpm)	64.31 (10.06)	71.3 (12.96)	3.62 (79.28)	<0.001	0.64	0.26, 0.88
Respiratory peak frequency (Hz)	0.29 (0.05)	0.29 (0.05)	−0.15 (88.71)	0.88	−0.02	−0.33; 0.29
PANSS totaI^b^	—	48.03 (12.77)				
Positive symptoms^b^	—	10.23 (3.85)				
Negative symptoms^b^	—	11.67 (4.88)				
General symptoms^b^	—	26.13 (6.42)				

Values are means with standard deviations in parenthesis. HC = Healthy controls; PSD = Psychosis spectrum disorder; BMI = Body mass index; bpm = beats per minute; PANSS = Positive and negative syndrome scale; HF-HRV = High frequency heart rate variability (log transformed); RMSSD = Root mean square of successive differences. ^a^HC group n = 112, PSD group n = 39. ^b^PSD group n = 39.

**Table 2:** Demographic, clinical, and cardiorespiratory variables for clinical groups.

Group	SCZ (n = 34)	BD (n = 21)	*t* (df)	*p*	Hedges’ *g*	95% CI
Age	30.38 (8.92)	29.9 (9.55)	0.18 (40.27)	0.85	0.05	−0.49; 0.6
BMI^a^	25.36 (3.1)	23.71 (3.6)	1.51 (31.68)	0.14	0.49	−0.16; 1.13
HRV	6.13 (1.13)	6.27 (1.19)	−0.46 (40.92)	0.65	−0.13	−0.67; 0.42
Heart rate (bpm)	72.33 (12.98)	69.64 (13.07)	0.74 (42.26)	0.46	0.2	−0.34; 0.75
RMSSD	3.54 (0.59)	3.61 (0.61)	−0.44 (41.27)	0.67	−0.12	−0.66; 0.42
Respiratory peak frequency (Hz)	0.29 (0.06)	0.29 (0.05)	−0.07 (48.42)	0.95	−0.02	−0.56; 0.52
PANSS totaI^b^	52.74 (13.6)	41.25 (7.67)	3.36 (35.76)	<0.01	0.97	0.4; 1.77
Positive symptoms^b^	11.48 (4.26)	8.44 (2.25)	2.89 (34.93)	0.01	0.83	0.26; 1.61
Negative symptoms^b^	13.35 (5.54)	9.25 (2.18)	3.21 (30.36)	<0.01	0.89	0.36; 1.72
General symptoms^b^	27.91 (6.88)	23.56 (4.79)	2.33 (37)	0.03	0.7	0.09; 1.41

Note. Values are means with standard deviations in parenthesis. SCZ = Schizophrenia spectrum disorders; BD = Bipolar spectrum disorders; BMI = Body mass index; bpm = beats per minute; PANSS = Positive and negative syndrome scale; HF-HRV = High frequency heart rate variability (log transformed); RMSSD = Root mean square of successive differences. ^a^SZ group n = 22, BD group n = 17. ^b^SZ group n = 23, BD group n = 16.

**Table 3:** Relationship between respiration and both HRV and HR.

		HF-HRV	RMSSD	HR
Patients (n = 55)	Pearson’s r	−0.29	−0.13	0.31
	p-value	0.03	0.34	0.02
	Upper 95% CI	−0.03	0.14	0.53
	Lower 95% CI	−0.52	−0.38	0.04
Controls (n = 149)	Pearson’s r	−0.04	0.1	−0.02
	p-value	0.6	0.23	0.77
	Upper 95% CI	0.12	0.25	0.14
	Lower 95% CI	−0.2	−0.06	−0.18

Note: HF-HRV = High frequency heart rate variability (log transformed); RMSSD = Root mean square of successive differences; HR = Heart rate.
